# Timed written picture naming in 14 European languages

**DOI:** 10.3758/s13428-017-0902-x

**Published:** 2017-05-24

**Authors:** Mark Torrance, Guido Nottbusch, Rui A. Alves, Barbara Arfé, Lucile Chanquoy, Evgeny Chukharev-Hudilainen, Ioannis Dimakos, Raquel Fidalgo, Jukka Hyönä, Ómar I. Jóhannesson, George Madjarov, Dennis N. Pauly, Per Henning Uppstad, Luuk van Waes, Michael Vernon, Åsa Wengelin

**Affiliations:** 1 Division of Psychology, Nottingham Trent University, Nottingham, UK; 2 Department of German Primary Education, University of Potsdam, Potsdam, Germany; 3 Faculty of Psychology and Educational Sciences, University of Porto, Porto, Portugal; 4 Department of Developmental Psychology and Socialization, University of Padova, Padova, Italy; 5 Université Côte d’Azur Division CNRS, BCL, Nice, France; 6 Department of English, Iowa State University, Ames, IA USA; 7 Division of Psychology, Department of Primary Education, University of Patras, Patras, Greece; 8 Department of Psychology, Sociology and Philosophy, University of León, León, Spain; 9 Department of Psychology, University of Turku, Turku, Finland; 10 Laboratory of Visual Perception and Visuomotor control, Faculty of Psychology, University of Iceland, Reykjavik, Iceland; 11 Department of Psychology, St. Cyril and St. Methodius University, Veliko, Tarnovo Bulgaria; 12 Norwegian Reading Centre, University of Stavanger, Stavanger, Norway; 13 Faculty of Applied Economics, University of Antwerp, Antwerp, Belgium; 14 Department of Swedish, University of Gothenburg, Gothenburg, Sweden

**Keywords:** Written production, Response time, Interkey interval, Picture naming, Word production, Language production

## Abstract

We describe the Multilanguage Written Picture Naming Dataset. This gives trial-level data and time and agreement norms for written naming of the 260 pictures of everyday objects that compose the colorized Snodgrass and Vanderwart picture set (Rossion & Pourtois in *Perception, 33,* 217–236, 2004). Adult participants gave keyboarded responses in their first language under controlled experimental conditions (*N* = 1,274, with subsamples responding in Bulgarian, Dutch, English, Finnish, French, German, Greek, Icelandic, Italian, Norwegian, Portuguese, Russian, Spanish, and Swedish). We measured the time to initiate a response (RT) and interkeypress intervals, and calculated measures of name and spelling agreement. There was a tendency across all languages for quicker RTs to pictures with higher familiarity, image agreement, and name frequency, and with higher name agreement. Effects of spelling agreement and effects on output rates after writing onset were present in some, but not all, languages. Written naming therefore shows name retrieval effects that are similar to those found in speech, but our findings suggest the need for cross-language comparisons as we seek to understand the orthographic retrieval and/or assembly processes that are specific to written output.

Picture-naming tasks, in which participants rapidly name everyday objects depicted in simple line drawings, are a staple of psycholinguistic research. As a tool for understanding language production, picture naming is valuable both because it provides insight into the processes by which single words are retrieved (see, e.g., the multiple studies reviewed by Levelt, Roelofs, & Meyer, 1999) and as part of experimental manipulations exploring language production above the word level (e.g., Griffin, 2001; Spalek, Bock, & Schriefers, 2010; Zhao & Yang, 2016).

The cognitive process underlying picture naming are generally understood to involve information cascading through four processes (Alario et al., 2004; Humphreys, Riddoch, & Quinlan, 1988): perceptual processing of the structure of the visual stimulus, activation of semantic information about the object that it depicts, retrieval of associated lexical items (e.g., the object’s name), and motor planning of the movements required for outputting the response. The time to name a picture depends on both the complexity of processing at each stage and the extent to which different processing levels result in the activation of competing candidate representations. Both complexity and competition result from an interaction between representations of the physical features of the stimulus picture, of the object that is depicted, and of the characteristics of the language spoken by the participant (we will assume that naming is in the mother tongue). For example, the *naming latency* (time between stimulus onset and output onset) for a picture of a duck will depend on, at least, (a) the complexity of the image, (b) the extent to which the picture is similar to the participant’s mental image of a duck (*image agreement*), (c) the number of possible names associated with the concept DUCK (*name agreement*), and (d) the length of the final output (number of syllables in spoken names, number of letter—and possibly syllables—in written names). Some of these features are unlikely to vary across languages. This is likely to be true for image complexity and, assuming a reasonable homogeneity of culture, image agreement. Name agreement and output length, however, will be language-dependent. From current data, the picture from the Snodgrass and Vanderwart (1980) set that is more or less universally named as “duck” in British English receives at least four different names in Swedish (*anka*, 47% of participants; *and*, 25%; *gås*, 10%; and *ejder*, 7%). In French, as in English, this picture gives high name agreement, but the name—*canard*—is phonologically (and orthographically) longer.

These language-specific effects cannot be ignored either when choosing experimental stimuli or when interpreting findings. Cross-language comparison can also shed light on basic language processes. Picture-naming latency norms are currently available in the following European languages: Bulgarian, Dutch, English, French, German, Hungarian, Italian, and Spanish, with data from studies of single languages (Cuetos, Ellis, & Alvarez, 1999; Dell’acqua, Lotto, & Job, 2000; Rossion & Pourtois, 2004; Severens, Van Lommel, Ratinckx, & Hartsuiker, 2005; Snodgrass & Yuditsky, 1996) and from one multiple-language study (Bates et al., 2003). These studies are, however, all of spoken naming. Until relatively recently, research exploring the cognitive processes underlying word production, and language production more generally, has ignored written production.^1^ This exclusive focus on speech is, we believe, unwarranted. Understanding of language production necessarily requires understanding of written production, because of its ubiquity. It is also unlikely that theories of speech production in general, and of spoken naming in particular, apply without modification to writing. This would occur for at least two reasons.

First, written output necessitates retrieval (or assembly) of orthographic representations. For words with straightforward grapheme–phoneme mappings, spelling by assembly—breaking words into their phonetic components and retrieving the associated graphemes—in principle is possible. However, there is considerable evidence that direct semantic → orthographic activation without phonological mediation also occurs (Bonin, 2002; Bonin, Fayol, & Peereman, 1998; Miceli & Capasso, 1997; Rapp, Benzing, & Caramazza, 1997; Sahel, Nottbusch, Weingarten, & Blanken, 2005): The concept DUCK can provide direct access to an associated orthographic lexeme (a language-dependent representation of the orthographic word—*canard*, *πάπια*, *duck*, etc.), without an intermediate stage involving retrieval of the word’s phonology. It seems unlikely, therefore, that written naming can be explained adequately simply by bolting orthographic retrieval onto existing models of spoken production as a process occurring after retrieval of the phonological word form. However, there is also evidence that processing from concept to orthographic lexeme is not informationally encapsulated and, under some circumstances, is influenced by phonology (Bonin & Fayol, 2000; Bonin, Roux, Barry, & Canell, 2012; Nottbusch, Grimm, Weingarten, & Will, 2005; Roux & Bonin, 2012; Zhang & Damian, 2010). For example, a word’s syllable structure, independently of digraph frequency, affects the time course of its production, with output slowing at syllable boundaries (Kandel, Álvarez, & Vallée, 2006; Kandel, Peereman, Grosjacques, & Fayol, 2011).

Spelling is therefore, in principle at least, a dual-route process (Barry, 1994; Martin & Barry, 2012). Spelling can be assembled from phonology or retrieved directly from an orthographic lexicon, and it is probably best understood as a race between these two processes (e.g., Paap & Noel, 1991). Even in the spelling of nonwords to dictation, in which retrieval of an intact lexical item is necessarily impossible, processing (in English speakers at least) does not seem to occur purely by sound-to-letter assembly, but is influenced by the orthographic lexicon (Martin & Barry, 2012). The relative roles of sound-to-letter assembly and direct orthographic retrieval are, however, likely to be language-dependent. European languages (or, strictly, writing systems) vary considerably in orthographic depth—that is, in the extent to which their words show regular sound-to-spelling mappings. Where words have predictable sound-to-spelling correspondences, then spelling by assembly is clearly more reliable. Delattre, Bonin, and Barry (2006) found that phoneme–grapheme mapping regularity, manipulated across items within a single language (French) predicted both production onset and writing speed in a written transcription task. Trivially, therefore, there is likely to be variation in these effects across languages simply because languages vary in the proportions of regularly spelled words that they contain. More fundamentally, it may be that learning to spell in a shallow orthography results in the development of spelling processes that are qualitatively different from those that result from learning to spell in, for example, English. Share (2008) has argued that accounts of single-word reading (spoken naming of words) have been skewed by an excessive focus on English, which has an unusually deep orthography. The written-naming literature is smaller, but it also is dominated by studies in English and in French (also a nontransparent orthography). This is further reason for cross-language comparisons.

A second reason why theories of spoken naming cannot be applied uncritically to written naming is that, unlike speech, writing decouples output fluency and communicational effect. Midword hesitations affect listeners’ inferences about a speaker’s intent, and possibly inhibit recognition of the word. This communicational pressure to be fluent is absent when output is written. Pausing midword (in most writing contexts) has no effect on how the word will be processed by its reader(s), regardless of pause duration. Relaxing the output fluency constraint means that there is potential for some processing to be deferred until after production onset. The study of written production therefore provides a direct test of the extent to which output planning can be achieved incrementally—whether or not the complete word form must necessarily be retrieved before output can commence—unconfounded by fluency constraints.

Written picture naming is, therefore, psychologically interesting but underresearched. In the present article we describe a study in which relatively large samples of adult speakers using each of 14 European languages gave typewritten names for the 260 pictures the of the colorized version of the Snodgrass and Vanderwart picture set (Rossion & Pourtois, 2004; Snodgrass & Vanderwart, 1980). The resulting Multilanguage Written Picture Naming Dataset will be of value to researchers for two reasons. First, it provides response agreement and timing norms that can be used as a basis for stimulus selection in future studies that involve written naming of pictures. Second, analysis of data from the existing dataset, which is publically available, has the potential to shed light on the cognitive processes underlying written word production both within specific languages and by making cross-language comparisons. This potential has been demonstrated by research exploring the effects of a range of word-level factors of production naming latency in just the Italian subset of the dataset (Scaltritti, Arfé, Torrance, & Peressotti, 2016) and by the preliminary analyses across all 14 languages reported in this article.

Written-naming tasks, like spoken naming, give measures of name agreement—the spread of different names generated in response to a particular picture stimulus—and of response latency (RT; the time from stimulus onset to typing onset). Additionally, written naming makes available information about *spelling agreement*—the extent to which the spelling given to an object’s name is consistent across participants—and about the production time course after output has been initiated (within-word writing rate). Spelling agreement for a particular picture is dependent in part on the spelling regularity of its name, which in turn will be language-dependent. In the present study, for example, there was very high agreement among participants about the name of the 52nd picture (a picture of a chain) in both the English and French samples. However, in English 95% of the participants gave the modal spelling (*chain*), as compared to only 62% in French (*chaine*). In spoken production, name agreement is a relatively strong predictor of RT for trials in which the participant provides the modal name. In other words, even in situations in which the response given by the participant is the same as that given by the majority of other participants, the possibility of alternative names increases the response latency. This effect holds true across a number of languages (e.g., Bates et al., 2003; Székely et al., 2003). In the analyses reported in this article, we tested the hypothesis that low name agreement also will be associated with longer RTs in written naming, and explored the possibility that spelling agreement would show similar effects.

Written naming also gives measures of the postonset (within-word) production time course. In spoken-naming studies, the time course of output after the participant has started to speak is not routinely analyzed (but see, e.g., Buz & Jaeger, 2016). This measurement is technically tricky and subject to the communicational pressure to be fluent that we described above. In written, and particularly in typewritten, production, obtaining information about the time course after writing onset is more straightforward. There is debate concerning which of the various processes associated with word retrieval and production must be complete prior to starting to write the word, and which can be postponed or completed after writing onset. Early models, focused on handwritten production, drew a strong distinction between the central processing associated with lexical retrieval, completed prior to output, and the peripheral motor processes required for response execution (Bonin, Peereman, & Fayol, 2001; Van Galen, 1991). Similarly, Crump and Logan (2010; Logan & Crump, 2011) argued, on the basis of evidence from typewritten production in English, for a two-process model, with an outer loop that generates word-level representations feeding an informationally encapsulated inner loop responsible for motor planning of the associated keystrokes. There is, however, evidence that lexical processing is not always complete at writing onset (or, more weakly, that inner-loop processing is not informationally encapsulated). This appears to be true in Finnish and Italian—orthographically shallow languages in which an incremental (letter-by-letter assembly) strategy is very likely to reliably generate correct spelling (Bertram, Tønnessen, Strömqvist, Hyönä, & Niemi, 2015; Scaltritti et al., 2016)—but also in French (Delattre et al., 2006; Kandel & Perret, 2015; Lambert, Kandel, Fayol, & Espéret, 2008; Roux, McKeeff, Grosjacques, Afonso, & Kandel, 2013) and English (Gentner, Larochelle, & Grudin, 1988).

The Multilanguage Written Picture Naming Dataset therefore provides name and spelling agreement norms, as well as trial-level response latencies and within-word (post-output-onset) time course data, for participants providing typewritten picture names in 14 alphabetic European languages. These languages vary in orthographic depth. Deviation from one-to-one phoneme–grapheme mapping can take many forms, which makes cross-language measurement of orthographic depth problematic. Borgwaldt, Hellwig, and De Groot (2005) provided one possible measure, in terms of the number of possible word-initial letter-to-phoneme mappings, although these data are only available for some of the languages that we sampled. Seymour, Aro, and Erskine (2003) provided a less formal though frequently cited classification. Combining these gives a very approximate orthographic-depth ranking of the languages that we sampled, as follows, starting with the most orthographically transparent: Finnish, Spanish, (Romanian), Italian, Icelandic, Norwegian, Portuguese, (Russian), German, Swedish, (Greek), Dutch, French, and English. Languages in parentheses are absent from both the Borgwaldt et al. and Seymour et al. classifications.

Our choice of typing, as opposed to handwriting, as the output modality was expedient rather than principled. Keystroke timing is easier to capture and to analyze than pen-stroke timing. Keystroke actions typically also have a clearer interpretation, in that they necessarily represent the endpoint of orthographic and motor planning processes associated with the current letter (whereas the pen-stroke duration can potentially be varied to accommodate additional processing). Early research focused on typing as a minority, specialized motor skill (e.g., Gentner, 1982; Logan, 1982; Rumelhart & Norman, 1982). Now, 25 years later, typing is still learned after handwriting, but it is reasonable to assume that university-level writers—the population sampled in this and many other naming studies—are at least “functionally competent” typists, with keyboarding as their dominant written output modality. Logan and Crump (2011), in a large survey of US college students, found average typing speeds of 68 words per minute and an average age of 10 years for when students started to type. Although US students may be particularly skilled—most report some formal training—we anticipate broadly similar skills in most European countries. Norway, for example, requires that all upper-secondary students own and use a laptop, and from 2017, Finnish primary schools are replacing the teaching of cursive handwriting with keyboarding instruction, although children’s first contact with writing is still via noncursive handwriting. Handwriting and typing clearly differ at the motor level. We are not aware, however, of evidence that suggests that these motor differences interact with upstream lexical and orthographic processing. Given that typing is now ubiquitous, we believe that it is a valid context in which to observe written-naming effects. It is worth noting, however, that handwriting on paper and typing at a computer have rather different affordances. Within-word errors are probably quicker to correct when typing and, more importantly, are then invisible to the reader. This may affect the speed–accuracy trade-off.

Our purpose in the remainder of this article is to describe our data collection and processing methods in sufficient detail for potential users to make informed decisions about the value of the Multilanguage Written Picture Naming Dataset to their research. We also present some preliminary analyses, with a focus on the effects of factors that are typically presented in studies reporting spoken picture-naming norms. Specifically, we describe the effects on RTs and the writing time course of picture-level factors (familiarity, complexity, image agreement), of name length and frequency, and of name and spelling agreement.

## Method

### Participants

The participants were undergraduate and postgraduate students recruited at universities in each of 14 European countries. Details are given in Table 1. All participants self-reported as competent typists and as not having first-language or literacy difficulties. In addition to demographic questions, we also asked participants about their own perceptions of their typing ability and about their typing habits. We asked which fingers participants used when typing and whether or not they used both hands, providing a number of specific options and an open question that participants could use if none of the given answers represented their behavior. We also asked where they looked when typing, with five options, ranging from always looking at the screen to always looking at the keyboard. Findings from these questions are also provided in Table 1.

**Table 1:** Participant details

Language	*N* (% Female)	Mean Age (*SD*)	Self-Reported Habitual Typing Behavior				Mean % Edit-Free Responses (*SD*)
			Ability (*SD*)	% 4 Fingers	% ≥6 Fingers	% Screen Gaze	
Bulgarian	81 (68%)	26.9 (8.2)	2.70 (1.39)	35	51	46	86.1 (7.14)
Dutch	60 (73%)	23.6 (13.7)	3.09 (1.59)	22	69	72	83.0 (12.1)
English	103 (76%)	22.1 (6.5)	3.27 (0.94)	31	59	41	84.6 (6.04)
Finnish	100 (61%)	24.4 (3.9)	3.12 (1.24)	25	70	72	85.2 (6.67)
French	100 (61%)	25.2 (6.2)	2.70 (1.10)	26	50	29	85.2 (8.45)
German	121 (72%)	26.1 (4.8)	2.84 (1.13)	38	53	29	86.3 (6.37)
Greek	102 (76%)	23.6 (4.5)	3.23 (1.42)	34	40	24	81.9 (7.31)
Icelandic	81 (62%)	30.4 (10.9)	3.09 (1.44)	8	88	69	81.7 (7.73)
Italian	82 (73%)	24.5 (3.4)	3.59 (1.30)	49	40	29	88.5 (6.60)
Norwegian	82 (75%)	29.0 (10.3)	3.20 (1.15)	29	64	49	86.8 (6.65)
Portuguese	81 (95%)	22.2 (5.7)	3.14 (1.37)	64	21	34	81.9 (7.26)
Russian	102 (49%)	22.3 (4.1)	3.01 (1.29)	36	49	22	89.5 (5.35)
Spanish	119 (89%)	24.1 (12.2)	3.50 (1.12)	25	69	39	82.8 (5.80)
Swedish	60 (86%)	27.3 (7.0)	2.73 (1.12)	25	68	39	87.0 (4.99)

Typing Ability: *Ability*, 1 = *very good* to 7 = *very poor. 4 fingers*, reports using at least two fingers on each of both hands. *≥6 fingers*, reports using most fingers on each of both hands. *Screen gaze*, reports looking at the screen mainly or all of the time when typing. A response to an image was *edit-free* if it included no delete (backspace) or cursor-move keystrokes. Trials that included editing were removed from the analyses reported in this article.

### Design, materials, and procedure

Participants saw each of the 260 pictures of common objects that compose the picture set originally created by Snodgrass and Vanderwart (1980) and subsequently colorized by Rossion and Pourtois (2004). Rossion and Pourtois redrew the pictures, keeping very close to the originals, and then added realistic color and texture. Participants gave names for these pictures, typing their responses on a computer keyboard. Participants were asked to name the picture in their first (dominant) language, writing whatever name they would normally give for the object and spelling the name as accurately as possible. We recorded both the response latency and the mean interkeypress interval. Responses were then coded to give measures of both name and spelling diversity.

The experiment was implemented within the SR Research Experiment Builder environment, with keypress (and release) times being accurately captured by in-house code described in Wengelin et al. (2009). Participants typed on computer keyboards with typical, language-specific layouts. Evidence from Damian (2010) suggests that, in the context of language production research, timing inaccuracies resulting from using standard keyboards as input devices are sufficiently small to be unlikely to compromise findings.

Participants were tested either individually or in groups (under “examination conditions”). They completed first ten practice trials and then four blocks of 65 trials, with the order randomized across trials and blocks. Trials started with a fixation point, displayed just above the center of the computer screen. This was replaced, after a random interval between 600 and 1,000 ms, by the stimulus picture. Pictures measured approximately 8 cm across their largest dimension. Participants’ typed responses appeared immediately below the image. They pressed the Enter key when they had finished typing their response, and then progressed to the next trial.

At the starts of both the practice trials and the main experiment, participants read (a translation of) the following text:

In this experiment you will see simple pictures on the screen. All you have to do is to type the name of the thing that is shown in the picture. So, for example, is you see a picture of a cat you will type *cat*. You should be both quick and accurate. Sometimes there might be more than one name that you could give to the picture. Just write the first that comes into your mind. If you make a mistake, you can use the backspace key to delete.^2^

Researchers gave spoken instructions repeating these instructions. They also told participants that they should give their best guesses in cases in which the name and/or spelling did not come easily to mind, and that names including more than one orthographic word were permitted (e.g., *rolling pin*).

### Measures

#### Timing

We recorded press times for each key pressed during the production of each response. We also recorded key release times; these are available within the dataset but are not considered further in this article. Where the generation of a character required the pressing of two or more keys—as is necessary, for example, in some keyboard layouts when typing some diacritics—we calculated the times to press both the modifying key (the first key in the set) and the final, character key. The RTs for the by-picture norms and the analyses reported in this article are based on the first of these two measures.^3^ RTs were timed from the picture’s appearance on the screen to first keypress. We calculated interkeypress intervals for all keypresses after the word-initial keypress (but excluding the final, trial-terminating Enter keypress). The interkeypress interval was defined as the interval between the press time for the current key and the press time for the immediately preceding key.

#### Name and spelling agreement

Naming and spelling agreement were established as follows: We first identified “null” responses: that is, responses that were not object names (e.g., *don’t know*; *xxx*; *thing*) or were blank. Then, within each language and for each picture, the remaining responses were grouped to give phonologically plausible spellings of the same name. For example, the following responses made by UK English speakers to a picture of an airplane were categorized as three different names: *plane*, *plaine*/*aeroplane*, *areoplane*, *airoplane*, *earoplane/airplane*, *air plane*. Additionally, the following were coded as spelling variants of the same name: inflectional variants, phrasal nouns varying only in word order, compounding variants (*airplane*, *air plane*), responses including punctuation, responses with and without an article, and responses with additional whitespace. On the basis of this coding, we identified the *modal name* as the name with the highest total frequency, summing across all variant spellings, and the *modal response*, which we defined as the most common spelling of the modal name (although, in principle at least, it would be possible for the true modal response to be a nonmodal name). In almost all cases, the modal response was the canonical, “dictionary” spelling.

We calculated six agreement measures, detailed in Table 2. *H-name* and *H-spell* are both based on the commonly used *H* index (Lachman, 1973). This provides a measure of response diversity based on the number of different responses to a picture, weighted by the frequency with which each alternative response was given. *H-name* is a measure of the dispersion of names given to a particular object. Low scores represent good cross-participant agreement in how a picture should be named. *H-spell* is a measure of the dispersion of the spellings of the modal name. Low scores indicate high agreement across participants in how this name was spelled. *Levenshtein distances* were calculated between each nonmodal spelling of a particular name and the associated modal spelling. We also calculated, for each picture, the percentage of participants giving the modal name and the percentage of participants who gave the modal spelling for the modal name. Finally, in the present article we also report for each language the number of pictures with 90% or greater agreement in spelling (i.e., participants giving the same spelling) for the modal name.

**Table 2:** Descriptive statistics for the variables derived in this study

	RT (ms)^a^	MIKI (ms)^a^	Name Agreement (H-name)^b^	Spelling Agreement (H-spell)^b^	Participants Giving Modal Name (%)	Participants Giving Modal Spelling for Modal Name (%)^c^	Levenshtein Distance^a,*d*^	Pictures With >90% Modal Spelling (%)^e^	Length of Modal Response (Letters)
Bulgarian	1436 (1312, 1629)	265 (236, 300)	.48 (.17, 1.07)	.27 (.18, .40)	93 (76, 98)	96 (94, 97)	1.00 (1.00, 1.25)	92	5 (4, 7)
Dutch	1097 (991, 1266)	150 (136, 160)	.47 (.12, 1.08)	.00 (.00, .16)	92 (72, 98)	100 (98, 100)	1.00 (1.00, 2.00)	91	5 (4, 7)
English	1127 (1026, 1248)	162 (148, 178)	.35 (.08, 1.01)	.22 (.08, .39)	95 (78, 99)	97 (95, 99)	1.00 (1.00, 1.33)	83	5 (4, 6)
Finnish	1096 (979, 1255)	154 (141, 170)	.60 (.19, 1.28)	.08 (.00, .16)	89 (66, 97)	99 (98, 100)	1.00 (1.00, 1.32)	97	6 (5, 8)
French	1258 (1152, 1390)	199 (187, 212)	.47 (.19, .93)	.62 (.37, 1.09)	93 (81, 97)	91 (82, 95)	1.13 (1.00, 1.44)	53	6 (5, 7)
German	932 (851, 1036)	208 (189, 230)	.39 (.07, 1.00)	.14 (.07, .24)	94 (75, 99)	98 (97, 99)	1.00 (1.00, 1.50)	92	6 (5, 7)
Greek	1589 (1370, 1776)	272 (252, 291)	.53 (.22, 1.22)	.54 (.44, .76)	92 (75, 97)	91 (87, 92)	1.07 (1.00, 1.17)	53	6 (5, 8)
Icelandic	1201 (1072, 1380)	171 (157, 187)	.50 (.17, 1.20)	.17 (.05, .29)	92 (73, 98)	98 (96, 99)	1.00 (1.00, 1.33)	89	6 (4, 7)
Italian	1261 (1134, 1427)	199 (186, 211)	.37 (.10, 1.00)	.10 (.00, .20)	94 (76, 99)	99 (97, 100)	1.00 (1.00, 1.00)	97	7 (5, 8)
Norwegian	1286 (1160, 1501)	209 (185, 234)	.47 (.10, 1.19)	.19 (.00, .35)	93 (71, 99)	98 (95, 100)	1.00 (1.00, 1.33)	84	5 (4, 7)
Portuguese	1260 (1102, 1466)	183 (168, 201)	.36 (.10, 1.08)	.19 (.10, .38)	95 (78, 99)	98 (95, 99)	1.00 (1.00, 1.25)	85	6 (5, 7)
Russian	1447 (1326, 1610)	256 (230, 280)	.54 (.16, 1.11)	.17 (.08, .33)	91 (74, 98)	98 (96, 99)	1.00 (1.00, 1.00)	90	5 (4, 7)
Spanish	1198 (1063, 1343)	178 (165, 205)	.39 (.12, .96)	.16 (.07, .40)	94 (82, 98)	98 (94, 99)	1.00 (1.00, 1.11)	84	6 (5, 7)
Swedish	1196 (1095, 1362)	182 (165, 206)	.33 (.00, .93)	.00 (.00, .13)	95 (80, 100)	100 (98, 100)	1.00 (1.00, 1.19)	96	5 (4, 7)

Median across items with lower and upper quartile bounds are in parentheses. ^a^Values for RT, MIKI, and Levenshtein distance are averages across pictures of by-picture values averaged across participants. ^b^Note that higher values represent lower agreement. ^c^For participants who gave the modal name, percent of participants who gave the modal spelling. ^d^Distance between the modal spelling and nonmodal spellings of the modal name. ^e^ Percent of pictures such that when the picture was given the modal name this was with the modal (correct) spelling in a least 90% of cases.

#### Name and image measures

We report *name length—*the number of letters in the modal response—and *name frequency*—the frequency of the modal name, taken from language-specific print corpora. We used surface-form frequencies for all languages except Finnish, for which we used lemmatized frequencies. Finnish nouns can be inflected in an exceptionally large number of ways, making the surface frequency a poor proxy for name familiarity. Full details of the frequency data sources can be found with the dataset at https://doi.org/10.6084/m9.figshare.4898144.v2.

We took by-image ratings of familiarity, complexity, and image agreement from the norms reported by Rossion and Pourtois (2004). *Familiarity* is a rating of how familiar the depicted object is to a participant, *complexity* is a rating of the visual complexity of the image,^4^ and *image agreement* of how closely the image matches the participant’s own mental representation of what the object looks like.

## The Multilanguage Written Picture Naming Dataset

The dataset resulting from the methods described above provides both raw, by-trial data and by-picture norms for each language. We will describe these separately.

### By-trial response data

The full dataset provides, for each trial, the response given by the participant (the final string as it appeared on the screen when the participant pressed Enter to end the trial) and the keypress sequence that led to this output. These will differ in cases in which the participant made but then corrected errors while typing. The keypress sequence therefore may include backspace and cursor-move keypresses. Where these were present, the response is flagged as *nonfluent*. For each keystroke, we give both the press and release (key-lift) times, and calculated the interkeypress intervals. This complete dataset therefore permits not just investigations of lexical retrieval and spelling processes, but also of processes associated with monitoring and correction and with the planning and generation of subword units.

### By-picture norms

We provide, by-picture, the following variables for each of the 14 language samples: modal response; frequency and length of the modal response; the five diversity measures described above; lists of the most frequent alternative names and the most frequent alternative spellings of the modal name, with the proportions of participants giving these responses; proportions of null responses; and mean RT and mean interkeypress interval (MIKI).

We provide separate mean RT and MIKI values for (a) just trials in which participants gave the most common spelling for the modal name, and (b) all trials with non-null responses. Before we calculated RT and MIKI, the data were screened by first removing all trials in which the response was nonfluent, on the grounds that in cases in which the response word was edited, the initial latency cannot be directly associated with preparation of the final response, and the MIKI becomes similarly difficult to interpret. This resulted in the removal of 15.0% of all trials, averaged across languages (*SD* = 2.5%), with some variation across languages (minimum [Russian], 10.5%; maximum [Icelandic], 18.3%; see Table 1, final column). Then, when calculating the modal-response RTs and MIKIs, we removed all trials in which participants did not give the modal response (*M* = 19.5% of remaining trials, *SD* = 3.3%). We then removed outliers on a by-language basis. Our approach (following that of Van Selst & Jolicœur, 1994) involved first calculating *SD*s across all trials with nonextreme values, and then removing from the full dataset (including extreme values) outliers that deviated more than three *SD*s from the mean. We repeated this process twice, first for RTs, with extreme values defined as <300 ms or >5,000 ms, removing 3.0% of trials (averaged across languages; *SD* = 0.6%), and then for MIKIs, with extreme values defined as >1,000 ms, removing an additional 1.5% of trials (*SD* = 0.2%). We followed the same data-trimming procedure when calculating the mean RTs and MIKIs across all responses, except that we did not remove nonmodal responses.

Table 2 gives by-language summary statistics, averaging across pictures. Both the time and agreement measures were negatively skewed, and this was particularly pronounced for H-name and H-spell. We therefore present medians and quartile boundaries, averaged across pictures (first taking the mean across participants for values that varied on a by-trial basis). The median percentage of null responses was either 0 or 1 for all languages except Russian (*Mdn* = 3). Table 3 reports correlations across pictures among the picture-specific variables, modal response length and frequency, and name and spelling agreement. The correlations among picture-specific variables (necessarily constant across languages) were as follows: familiarity–complexity = –.54; familiarity–image agreement = –.11; image agreement–complexity = .00 (Pearson *r*).

**Table 3:** Correlations across pictures among measures of image familiarity, complexity, image agreement, and H-name, H-spell, and modal name length

	Fam	Comp	IA	Hnm	Hsp	Len	Fam	Comp	IA	Hnm	Hsp	Len
	Bulgarian						Dutch					
H–name	–.18	.08	–.23				–.17	.04	–.19			
H–spell	–.02	.02	–.05	.04			–.14	–.02	.05	–.03		
Length	–.20	.05	.06	.02	.27		–.21	.15	.22	.13	.21	
Frequency	.43	–.13	–.20	–.08	–.19	–.35	.42	–.15	–.18	–.31	–.13	–.47
	English						Finnish					
H–name	–.21	.12	–.08				–.09	.06	–.24			
H–spell	–.25	.16	.08	.20			–.02	.05	.01	–.03		
Length	–.18	.11	.17	.12	.60		–.15	.04	.20	.07	.25	
Frequency	.47	–.16	–.24	–.25	–.44	–.54	.34	–.02	–.13	–.29	–.18	–.59
	French						German					
H–name	–.17	.14	–.25				–.17	.06	–.20			
H–spell	–.25	–.03	.08	–.02			–.14	.11	.20	.06		
Length	–.14	.05	.08	–.01	.31		–.13	.02	.12	.07	.40	
Frequency	.56	–.19	–.27	–.11	–.43	–.35	.35	–.05	–.23	–.10	–.31	–.45
	Greek						Icelandic					
H–name	–.21	.17	–.22				–.21	.10	–.10			
H–spell	–.10	.15	.09	.11			–.07	.09	.05	.00		
Length	–.11	.13	.07	–.09	.27		–.08	.05	.17	.04	.42	
Frequency	.40	–.13	–.18	–.17	–.27	–.33	.34	–.12	–.20	–.21	–.24	–.50
	Italian						Norwegian					
H–name	–.14	.14	–.30				–.21	.03	–.12			
H–spell	.04	–.08	–.04	.15			–.03	.05	.04	.01		
Length	–.04	.01	–.05	.05	.29		–.15	.02	.14	.23	.43	
Frequency	.55	–.19	–.18	–.17	–.12	–.27	.32	–.11	–.14	–.34	–.25	–.56
	Portuguese						Russian					
H–name	–.37	.20	–.04				–.11	.04	–.22			
H–spell	–.08	.04	.01	.13			–.03	.04	–.04	.06		
Length	–.15	.16	.05	.20	.27		.00	.00	.09	–.07	.27	
Frequency	.43	–.15	–.16	–.33	–.21	–.37	.47	–.08	–.19	–.11	–.16	–.24
	Spanish						Swedish					
H–name	–.31	.12	–.11				–.14	.03	–.19			
H–spell	–.02	.03	.11	–.06			.04	–.05	.08	–.02		
Length	–.12	.04	.04	.05	.33		–.15	.05	.09	.17	.28	
Frequency	.24	.02	–.11	–.25	–.17	–.40	.44	–.13	–.16	–.30	–.15	–.50

Spearman rank correlations. Fam = Familiarity, Comp = Complexity, IA = Image agreement, Hnm = H-name, Hsp = H-spell, Len = name length in letters. Length and frequency are for the modal name. *p* < .001 for |*r*_s_| ≥ .18.

### Effects of the picture and name variables on RT and production rate

Our aim in the analyses that follow was to determine, separately for each language, the extent to which the RT and MIKI were predicted by picture familiarity, complexity and agreement, name frequency length and agreement, and spelling agreement. We analyzed only trials in which the participant gave the modal response, with data trimmed as detailed above.

First we report incremental mixed-effects regression analyses with familiarity, complexity, image agreement, and name frequency and length as predictors. We then report separate analyses of the effects of H-name and H-spell.

### Familiarity, complexity, image agreement, length, and frequency effects

We compared four incremental models, starting with a zero model with random by-picture and by-subject intercepts and random by-subject slopes for familiarity, image agreement, and frequency.^5^ We then added the picture-related factors (familiarity, complexity, image agreement; Model 1), then name length (Model 2), and finally name frequency (Model 3). Model fits were compared on the basis of *χ* ^2^ change, and individual coefficients were evaluated against a *z* distribution. Prior to the analysis of frequency, RT and MIKI were log-transformed, and all predictor variables were standardized (within language).

Table 4 gives retransformed coefficients from the final models (Model 3). For RT, Model 1 gave an improved fit over the zero model for all languages [across all language, *χ* ^2^(3) > 35, *p* < .001]. Name length gave additional, statistically significant effects in Dutch, Finnish, German, Icelandic, Norwegian, Portuguese, Spanish, and Swedish [*χ* ^2^(1) > 9.0, *p* < .003], but not in Bulgarian, English, French, Greek, Italian, and Russian. Adding frequency improved the fit in all languages [Dutch, Norwegian, German, Portuguese, and Swedish, *χ* ^2^(1) > 4.0, *p* < .05; other languages, *χ* ^2^(1) > 13, *p* < .001]. The final model showed effects of familiarity and image agreement in all languages, with quicker responses for more familiar objects and for pictures rated as having a good match to participants’ mental images. In all languages, participants generated higher-frequency names more quickly (controlling for name length, image agreement, and object familiarity).

**Table 4:** Extent to which ratings of image complexity, agreement with mental representation, and familiarity, as well as the length and frequency of the most common name, predict response latency and interkeypress interval

	Response Latency (RT)					Mean Interkey Interval (MIKI)				
	Familiarity	Complexity	Image Agreement	Length	Frequency	Familiarity	Complexity	Image Agreement	Length	Frequency
Bulgarian	–73 (–100, –43)^**^	–22 (–49, 10)	–75 (–95, –52)^**^	12 (–14, 42)	–67 (–91, –40)^**^	–1 (–6, 5)	–2 (–7, 3)	1 (–3, 6)	–1 (–5, 4)	–4 (–8, 2)
Dutch	–47 (–67, –25)^**^	–8 (–29, 16)	–51 (–68, –34)^**^	43 (21, 66)^**^	–24 (–41, –5)^*^	0 (–2, 2)	0 (–2, 3)	–1 (–2, 1)	3 (1, 5)^*^	–1 (–2, 1)
English	–49 (–70, –26)^**^	1 (–20, 23)	–31 (–47, –14)^**^	5 (–14, 25)	–46 (–65, –25)^**^	–1 (–3, 2)	2 (–1, 4)	3 (1, 5)^*^	4 (2, 6)^**^	–4 (–6, –2)^*^
Finnish	–57 (–76, –36)^**^	7 (–15, 30)	–34 (–50, –16)^**^	29 (8, 51)^*^	–54 (–71, –36)^**^	–1 (–3, 1)	0 (–3, 2)	0 (–2, 2)	2 (0, 5)^*^	0 (–2, 2)
French	–45 (–67, –21)^**^	11 (–11, 35)	–58 (–73, –41)^**^	1 (–18, 20)	–55 (–72, –35)^**^	1 (–2, 4)	4 (1, 7)^*^	–1 (–3, 1)	–2 (–4, 0)	–5 (–7, –3)^**^
German	–46 (–61, –30)^**^	1 (–16, 20)	–34 (–47, –20)^**^	18 (3, 34)^*^	–16 (–31, 0)^*^	–1 (–4, 4)	3 (–1, 7)	1 (–2, 5)	–3 (–6, 0)	–1 (–4, 3)
Greek	–82 (–115, –45)^**^	–10 (–44, 28)	–68 (–95, –40)^**^	–26 (–55, 6)	–70 (–100, –36)^**^	1 (–2, 5)	6 (3, 10)^**^	0 (–3, 3)	1 (–2, 4)	–2 (–5, 1)
Icelandic	–54 (–76, –29)^**^	2 (–22, 28)	–28 (–47, –7)^*^	22 (–1, 48)	–61 (–82, –38)^**^	0 (–2, 4)	2 (–1, 5)	0 (–3, 2)	3 (1, 6)^*^	–4 (–6, –1)^*^
Italian	–35 (–61, –7)^*^	10 (–14, 36)	–60 (–77, –42)^**^	1 (–20, 22)	–63 (–85, –39)^**^	–1 (–4, 1)	3 (1, 5)^*^	0 (–1, 2)	–2 (–3, 0)	–4 (–6, –2)^**^
Norwegian	–72 (–95, –47)^**^	–14 (–39, 12)	–25 (–45, –3)^*^	47 (20, 76)^**^	–45 (–69, –18)^*^	1 (–2, 5)	3 (–1, 7)	2 (–1, 6)	2 (–2, 5)	–6 (–9, –2)^*^
Portuguese	–55 (–92, –14)^*^	27 (–12, 71)	–43 (–72, –11)^*^	35 (2, 73)^*^	–62 (–94, –27)^**^	1 (–3, 4)	3 (0, 7)	0 (–2, 3)	1 (–1, 4)	–3 (–5, 1)
Russian	–56 (–82, –27)^**^	–6 (–32, 23)	–56 (–76, –35)^**^	6 (–17, 30)	–48 (–70, –23)^**^	–3 (–8, 2)	–1 (–5, 5)	–1 (–4, 4)	–1 (–4, 3)	1 (–3, 5)
Spanish	–82 (–102, –60)^**^	–9 (–32, 16)	–42 (–59, –23)^**^	15 (–6, 37)	–46 (–66, –25)^**^	0 (–3, 4)	4 (0, 7)^*^	1 (–1, 4)	2 (–1, 5)	–3 (–6, 0)
Swedish	–48 (–71, –24)^**^	7 (–16, 33)	–43 (–60, –24)^**^	40 (17, 65)^**^	–35 (–58, –10)^*^	1 (–3, 4)	1 (–2, 5)	1 (–1, 4)	4 (0, 7)^*^	–4 (–7, 0)^*^

Estimates are for change (in milliseconds) resulting from a 1 *SD* increase in the predictor. 95% CI in parenthesis. ^*^ *p* < .05, ^**^ *p* < .001. A 1 *SD* change in name length is approximately two letters.

By contrast, there were limited effects of these factors on production rate once output had been initiated: For MIKI, Model 1 gave significantly improved fits over the zero model for just English, French, Greek, Italian, and Spanish [*χ* ^2^(3) > 8.6, *p* < .035]. With the exception of English, these effects were associated with small positive effects of complexity (Table 4). Adding name length (Model 2) improved fit in Dutch, English, Icelandic, and Swedish [*χ* ^2^(1) > 15, *p* < .001], and also in Finnish, Norwegian, and Spanish, but with weaker effects [*χ* ^2^(1) > 5.8, *p* < .016]. Finally, frequency showed effects in just English, French, Icelandic, Italian, Norwegian, and Swedish [*χ* ^2^(1) > 4.6, *p* < .031], with higher-frequency words being written slightly more quickly.

### Name and spelling agreement effects

Rather than include H-name and H-spell as continuous predictors in the previous analysis, we conducted a separate analysis based on subsets of pictures with extreme high and low *H* values. This approach was necessitated by the fact that the data for some (but not all) languages included a large number of pictures for which there was zero name and/or spelling agreement, making transformation to a normal distribution misleading and, for some languages, impossible. It also emulated a stimulus selection strategy likely to be adopted by future researchers using these data.

On a by-language basis, we identified sets of 25 pictures in each cell of a 2 × 2 Name Agreement (high vs. low) × Spelling Agreement (high vs. low) design. Sets were selected so as to maximize low–high differences in *H* values within a language, keeping the mean values constant across levels of the other factor. Name length was controlled, with length varying by not more than 0.3 letters across cells within a language. The mean length across languages varied between 6.9 and 8.5 (*M* = 7.6, *SD* = 0.47). Item choice was algorithmic (i.e., researchers were blind to items during selection) to avoid unintentional bias (see Forster, 2000).

**Fig. 1:**
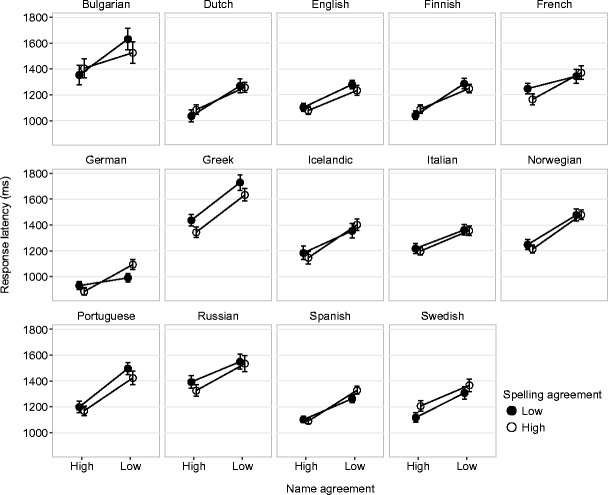
Observed mean response latencies by name agreement and spelling agreement. Error bars represent by-subject 95% confidence intervals (CIs)

**Fig. 2:**
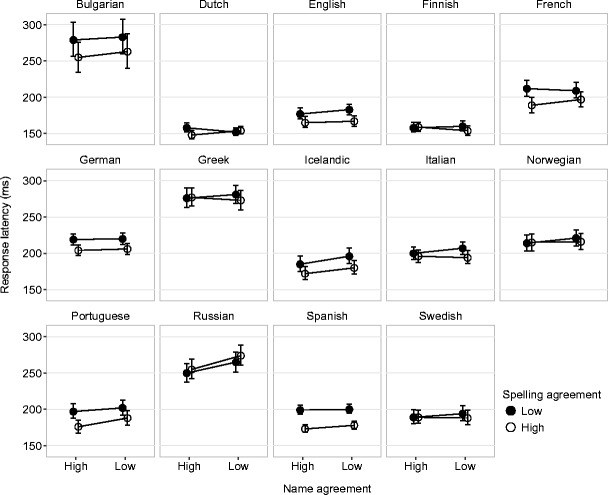
Observed mean interkeystroke latencies by name agreement and spelling agreement. Error bars represent by-subject 95% confidence intervals (CIs)

The observed mean RTs and MIKIs for these sets of pictures are given in Fig. 1 (RT) and Fig. 2 (MIKI). We tested separate linear mixed-effects models for each language, starting with a zero model with random by-picture and by-subject intercepts and random by-subject slopes for the main and interaction effects. We then added fixed effects of the categorical Name Agreement and Spelling Agreement factors and their interaction. RTs and MIKIs were log-transformed before the analysis. Coefficients from this final model are given in Table 5.

**Table 5:** Estimated effects of high naming and high spelling agreement (in milliseconds)

	Response Latency (RT)			Mean Interkey Interval (MIKI)		
	Name Agreement, Main Effect	Spelling Agreement, Main Effect	Interaction	Name Agreement, Main Effect	Spelling Agreement, Main Effect	Interaction
Bulgarian	.04 (.01, .07)^*^	–.02 (–.05, .02)	.04 (.00, .09)	.01 (–.02, .04)	.04 (.01, .07)^*^	–.01 (–.06, .04)
Dutch	.06 (.03, .10)^**^	–.02 (–.06, .01)	.04 (–.02, .09)	.02 (.00, .04)	.03 (.01, .06)^*^	–.03 (–.06, .00)
English	.05 (.02, .09)^*^	.01 (–.02, .04)	.02 (–.03, .07)	.00 (–.03, .03)	.03 (.00, .05)	.02 (–.02, .05)
Finnish	.07 (.03, .10)^**^	–.02 (–.06, .02)	.03 (–.02, .08)	–.01 (–.04, .01)	–.01 (–.03, .02)	.02 (–.02, .05)
French	.07 (.04, .10)^**^	.04 (.01, .07)^*^	–.04 (–.08, .00)	.02 (–.01, .04)	.04 (.02, .07)^**^	–.02 (–.05, .02)
German	.08 (.05, .11)^**^	.02 (–.01, .05)	–.05 (–.10, –.01)^*^	.00 (–.03, .03)	.02 (.00, .05)	.00 (–.04, .05)
Greek	.08 (.05, .12)^**^	.03 (–.01, .06)	.00 (–.05, .05)	–.01 (–.03, .01)	.00 (–.03, .02)	.02 (–.01, .05)
Icelandic	.09 (.05, .13)^**^	.01 (–.03, .05)	–.03 (–.08, .02)	.02 (–.01, .04)	.03 (.00, .06)^*^	.00 (–.04, .04)
Italian	.06 (.03, .09)^**^	.00 (–.03, .04)	.00 (–.05, .04)	.00 (–.02, .01)	.01 (–.01, .03)	.02 (–.01, .04)
Norwegian	.08 (.05, .12)^**^	.02 (–.01, .05)	–.02 (–.06, .03)	.01 (–.02, .04)	.01 (–.03, .04)	.00 (–.04, .05)
Portuguese	.09 (.05, .13)^**^	.01 (–.03, .05)	.02 (–.04, .07)	.03 (.00, .05)^*^	.05 (.02, .08)^**^	–.02 (–.06, .02)
Russian	.06 (.03, .09)^**^	.02 (–.01, .05)	–.01 (–.06, .03)	.03 (.01, .06)^*^	.00 (–.03, .03)	–.02 (–.06, .03)
Spanish	.09 (.06, .12)^**^	.00 (–.03, .03)	–.02 (–.07, .02)	.01 (–.02, .03)	.05 (.02, .08)^**^	.00 (–.04, .04)
Swedish	.05 (.02, .08)^*^	–.04 (–.07, –.01)^*^	.02 (–.02, .07)	–.01 (–.04, .02)	–.01 (–.04, .02)	.03 (–.02, .07)

95% confidence intervals in parentheses. Estimates are parameters from linear mixed-effects models with naming agreement (low *H* vs. high *H*) and spelling agreement (low *H* vs. high *H*) as categorical independent variables and log_10_ of the word-initial and mean interkeypress latencies as dependent variables, conducted separately for each language. ^*^ *p* < .05, ^**^ *p* < .001.

For RT, adding agreement effects to the model gave significantly and substantially better fits in all languages [*χ* ^2^(3) > 20, *p* < .001]. As can be seen from Table 5, this was associated almost exclusively with an increase in RTs for lower-name-agreement pictures. In French we found some evidence of an increase in RT as a result of lower spelling agreement. Swedish showed the reverse effect, although it should be noted that in Swedish spelling agreement (i.e., spelling accuracy) was generally very high across all pictures, with a mean H-spell of just .24 in the low-agreement condition. Contrast this with H-spell = .62 across all items in French (Table 2). We found evidence of an interaction only in German, with an underadditive effect of combining low name and spelling agreement.

For MIKI, adding agreement effects to the model improved the fit in only seven of the 14 languages (Bulgarian, English, French, Icelandic, Italian, Portuguese, and Spanish) [*χ* ^2^(3) > 9.3, *p* < .025]. Table 5 suggests weak evidence of name agreement effects persisting beyond typing onset for just Russian and Portuguese, with slower typing when the name agreement was lower. There were effects of spelling agreement in some, but not all, languages. Spanish, Portuguese, French, and Bulgarian showed quite large effects, with lower-spelling-agreement names being around 25 ms slower to type per letter than names with high spelling agreement. Dutch and Icelandic showed similar but smaller effects. The interaction between spelling and name agreement was not statistically significant in any language.

## Discussion

We believe that the description of methods and the preliminary analyses presented in this article demonstrate the potential value of the Multilanguage Written Picture Naming Dataset. It provides, for the first time, written picture-naming norms in a large number of languages, permitting informed choices of stimuli for future experiments. The dataset itself also permits direct testing of various hypotheses about lexical retrieval and spelling processes.

Our analyses indicate effects that are similar to those found in spoken naming: Name agreement, name frequency, image agreement, and image familiarity all predicted RTs in all 14 of the languages that we sampled. Spelling agreement, a factor that is clearly absent in spoken production, affected the production time course in some but not all languages—and this effect persisted beyond typing onset. Variation in effects across languages points to the need for cross-language triangulation before drawing strong conclusions about fundamental (language-independent) psycholinguistic processes. This appears true even amongst the exclusively alphabetic languages that we sampled in this study. Beyond these general conclusions, we offer the following specific observations.

Name agreement, averaged across pictures, showed relatively little cross-language variation. Comparison with spoken naming in the two existing comparable datasets suggests roughly similar agreement in Russian (*Mdn* H-name = .54 vs. .64 from Tsaparina, Bonin, & Méot, 2011) and higher agreement in French (.47 vs. .00 from Rossion & Pourtois, 2004). Without further data it is not clear, therefore, whether name agreement is different in spoken and written production. Conversely, and predictably, spelling agreement showed considerable variation across languages. This is seen most starkly when comparing the proportions of pictures for which the modal name, when given, was spelled correctly: In Finnish—a language with very regular phoneme–grapheme mappings—this was true for 97% of pictures. In French and Greek, this dropped to 53%. Spelling in French orthography is made particularly difficult by its one-to-many phoneme–grapheme mappings, meaning that the sound of a word often underdetermines how it will be spelled. This is also true in Greek. Additionally, Greek requires considerable use of diacritics to denote stress, and these are generated via a two-keystroke action. At minimum, these finding points toward the need to account for the kinds of language-specific differences in interpreting findings for written-naming studies.

Correlations between naming and spelling diversity (H-name and H-spell), and between these measures and picture complexity, familiarity, and agreement, tended to be very weak. In languages other than French, the latter finding could potentially be attributed to the fact that these picture-level ratings were taken from an earlier French sample and may not generalize to other samples from other countries. However, except for a small number of pictures, it seems unlikely that these ratings showed much language- or culture-specific variation within the present sample (European university students). This argument is also not consistent with relatively strong correlations between familiarity ratings and frequency (Table 3) and with effects of both familiarity and image agreement on RTs (Table 4) in all languages. We conclude, therefore, that for written naming both H-name and H-spell have good discriminant validity, indexing distinct underlying constructs that are not tapped by ratings of picture familiarity, complexity, and image agreement.

Direct comparison of the present written-naming RTs with spoken-naming latencies for colored Snodgrass and Vanderwart (1980) pictures is possible only for French: Rossion and Pourtois (2004) reported a median RT across all items of 844 ms. This compares with 1,258 ms for the French sample in this study, suggesting a time penalty of 414 ms for written naming. RT norms for the original black-and-white picture set are available for British English and American English (Barry, Morrison, & Ellis, 1997; Snodgrass & Yuditsky, 1996). Rossion and Pourtois found an 80-ms advantage for colored images over the original black-and-white line drawings. After we adjusted by this value, comparison of the means for the English sample in the present study with these spoken norms suggested roughly similar additional time costs of producing typewritten output (444 ms relative to British norms; 367 ms relative to American norms). The difference between spoken and written naming RTs will, in part, be due to the motor planning associated with preparing the first and perhaps subsequent keypresses, relative to the motor planning associated with articulation. The remainder can be attributed to the additional (or alternative) costs associated with orthographic processing.

Bates et al. (2003) found shorter spoken-naming RTs for pictures with high image agreement, for pictures with high-frequency names, and for pictures with high name agreement, but they did not find effects of picture complexity. These effects were present in all of the languages that they sampled. Our results suggest that the same holds true for written naming. However, effects of spelling agreement on RTs were largely absent. Several explanations are possible. First, for some languages, spelling accuracy was very high, and therefore differences between the high- and low-agreement pictures were small. However, this is at best a partial explanation, because some languages with large differences between high- and low-agreement items (Greek, English) also failed to show effects. We suggest two further explanations: First, spelling errors may sometimes occur as results of motor planning or execution errors, particularly in keyboarded production (i.e., are “typos”), rather than from problems at an abstract orthographic level (i.e., failure to retrieve or assemble the correct orthographic representation). These local motor errors are not likely to affect the latency prior to typing onset. Second, and more fundamentally, explanations for the effects of name agreement on RT transfer do not transfer straightforwardly to spelling agreement. Name competition explanations for slowed RTs rely on the assumption that even when participants give the most common name, their mental lexicon also contains alternatives. Retaining alternative names gives obvious benefits: An English speaker who always names a television “TV” nonetheless needs to retain “television” to allow comprehension of others’ discourse. The same is not true for spelling. Published text very rarely contains noncanonical spellings, and where it does this can typically be disambiguated by context or via grapheme–phoneme assembly. There is no benefit in retaining noncanonical (i.e., incorrect) orthographic lexical representations.

The MIKIs after typing onset showed quite a different pattern of results. Here there was little evidence of name agreement effects persisting into production of the word. This was to be expected, since it seems probable that conflict between different possible names must be complete before typing commences. This would hold true regardless of whether conflict occurs between modality-free (lemma-level) or modality-specific (phonological or orthographic) name representations (but see Scaltritti et al., 2016, for a possible account of effects of competitor names after typing onset). By contrast, in some but not all of the languages that we sampled, spelling agreement gave clear within-word effects, with lower-agreement names taking longer to write. Note that this effect is for trials in which the response was the most common name with the most common spelling and was produced without editing. So where a name had a tendency toward incorrect spelling, it was written more hesitantly even when the end result was correct. This suggests that, for some words in some languages, spelling is not fully retrieved before typing onset. There was evidence of this effect in French, for example—a finding consistent with results from studies of French handwritten production (Delattre et al., 2006; Roux et al., 2013). Several accounts are possible. One possibility is that this occurs particularly in languages in which an incremental phoneme → grapheme assembly strategy is often effective (Spanish, Portuguese), or at least gives no benefit over whole-word retrieval (contrast English and French), but where the language includes a number of ambiguous (one-to-several) phoneme–grapheme mappings (e.g., consonant doubling). Testing this hypothesis requires analysis of individual keystroke latencies within words with low spelling agreement. Rønneberg and Torrance (2017), for example, found that in a sample of 12-year-old children writing in a language with a shallow orthography (Norwegian), hesitation when correctly spelling irregular words tended to come immediately prior to typing the irregularity rather than prior to typing onset. Similar analyses will be possible within the Multilanguage Written Picture Naming Dataset.

The summary statistics and preliminary analyses reported in this article suggest a number of issues and questions about the effects of factors at the participant, lexical, and sublexical levels that might fruitfully be foci for future research. At the participant level, typing competence will affect performance. As we noted in our introduction, there has been a tendency in research exploring typed production to focus on typing skill and to sample highly skilled typists. This focus is absent in research exploring handwritten production, although handwriting processes probably show similar or greater cross-writer variation. Clearly, less skilled typists will give slower RTs and interkeypress intervals. However, it may also be that competence interacts with other predictors. Establishing typing skill is not straightforward. As we have discussed, the MIKI, as a possible measure of typing ability, is likely to index a combination of motor and orthographic skill, at least in some languages. On the basis of the self-report measures that we collected for this study, the Portuguese participants were furthest from the ten-finger, screen-gazing gold standard. However, they showed the 6th fastest mean MIKI across languages, with a value only 6 ms slower than typists who were specifically selected as skilled by Logan, Miller, and Strayer (2011), and they were not noticeably different from typists in other languages in their patterns of image and lexical effects. We repeated the analyses reported in Table 4 across all languages for just those participants who reported using three or more fingers and screen-gazing for at least half of the time spent typing, and this gave no substantive change in the pattern of results.^6^ We believe, therefore, that our argument that European university students are “functionally competent” typists is sustained by our findings. There is, however, considerable scope for exploring typing-skill effects within our dataset, based either on the writers’ self-report measures or on within-word interkeypress intervals, perhaps across a subset including only regular words and controlling for digraph and trigraph frequency.

The analyses reported in this article examined the effects of a relatively small subset of lexical factors and wholly ignored possible effects of sublexical (within-word) structure. Future analyses of the dataset might usefully explore age-of-acquisition effects, for example. These are present in written naming in French (Bonin, Méot, Lagarrigue, & Roux, 2015; Perret, Bonin, & Laganaro, 2014) and in the Italian subset of the present data (Scaltritti et al., 2016). Exploring the effects of sublexical factors gives insight into the detail of motor-planning process and scope and of an how these interact with upstream (lexical, phonological) processes. Transitions between keys vary, at least, in whether they cross a syllable boundary (cf. Kandel et al., 2006; Kandel et al., 2011, in handwritten production), whether they are before or within a high- or a low-frequency digraph or trigraph, whether the letter or letter group shows a regular phoneme–grapheme mapping (cf. Delattre et al., 2006, again in handwriting), and whether keypresses are with fingers on the same or on different hands (e.g., Rumelhart & Norman, 1982). The by-keystroke version of the dataset permits comparisons of these effects across languages, as well as control for these effects when choosing stimuli for other purposes.

In conclusion, our findings indicate that written picture naming shows many of the same effects as spoken naming, and that this holds true across a range of languages. One implication is that experiments that explore basic (and putatively modality-independent) language processes and that rely on manipulations involving picture stimuli (e.g., research exploring planning scope in sentence production) might usefully elicit written output alongside, or as a replacement for, speech. Second, differences in effects across languages, particularly in the effects of factors relating to spelling, indicate the importance of cross-language triangulation and comparison before making general (cross-language) claims about the underlying orthographic retrieval processes. With this in mind, we believe that the Multilanguage Written Picture Naming Dataset will support and motivate interesting future research.

## Footnotes

1 Here and throughout, we use the terms *writing* and *written production* to refer to the psycholinguistic processes necessary for the production of text, as opposed to speech, with no commitment to writing by hand or writing by keyboard (typing). The dataset that we report is derived from writing by keyboard. We discuss this choice of output modality below.

2 Note that trials that involved editing were removed from the analyses reported in this article.

3 This is based on the assumption that the initial keystroke in a pair (or, very rarely, a triple) is necessarily planned in combination with its partners and then executed as a chunk. The one general case that this assumption is likely to break down is for noun capitalization in German, in which the initial press of the Shift key for each response is entirely predictable without reference to the stimulus and could potentially be executed independently of planning the initial character. This may explain why initial latencies were shorter in German than for other languages, and it suggests that, for German, findings related to initial latency effects need to be treated with caution.

4 Following Székely and Bates (2000), we also looked at effects of the size, in bytes, of the image GIF file as an objective measure of visual complexity. This analysis gave substantively identical findings and is not reported here.

5 The length and complexity by-subject slopes made either no or very little contribution to the model fits.

6 A more stringent criterion—requiring the use of all fingers and more screen-gazing—gave insufficient sample sizes for some languages.
